# Fatigue have impact on the sexual problems in Chinese females with systemic lupus erythematosus

**DOI:** 10.1186/s12905-022-01854-3

**Published:** 2022-06-29

**Authors:** Lijuan Zhang, Beiwen Wu, Junna Ye

**Affiliations:** 1 Department of Nursing, Ruijin Hospital, Shanghai Jiao Tong University School of Medicine, 197, Ruijin Er Road, Shanghai, 200025 China; 2 Department of Rheumatology and Immunology, Ruijin Hospital, Shanghai Jiao Tong University School of Medicine, 197, Ruijin Er Road, Shanghai, 200025 China

**Keywords:** Chinese patients, Female, Systemic lupus erythematosus, Female sexual problems

## Abstract

**Background:**

Systemic lupus erythematosus (SLE) might affect all aspects of life including sexual function; previous study indicated that fatigue was the risk factor of sexual dysfunction. The current study aims to investigate the effects of SLE on Chinese mainland female patients’ sexual problems compared with healthy subjects and to investigate the relationship among fatigue, disease parameters, depression, quality of life and sexual problems in Chinese female patients with SLE.

**Methods:**

A total of 128 female SLE patients (mean age: 43.65 ± 7.13 years) and 121 healthy female controls (mean age 43.59 ± 6.57 years) were included in this cross-sectional study. All data were collected consecutively by face-to-face questionnaires from January 2021 to December 2021. SLE patients completed questionnaires for demographic or clinical variables, the 10-cm Visual Analog Scale for pain, the Systemic Lupus Erythematosus Disease Activity Index (SLEDAI) for disease activity, the multidimensional fatigue inventory (MFI) for fatigue, the patient health questionnaire-9 (PHQ-9) for depression, the Female Sexual Function Index (FSFI) for problems, and the Short Form 12 health survey for quality of life. Independent sample t-test, Mann–Whitney U-test, Chi-square test, and forward stepwise binary logistic regression model were used to analyze these data.

**Results:**

Our results showed that the prevalence of female sexual problems was 78.9% in SLE patients, which was significantly higher than the controls (56.7%; *p* < 0.05). The results found that having child (OR 23.04; *p* < 0.000), age (OR 1.11; *p* = 0.002), DMARDs usage (OR 0.04; *p* = 0.004), MFI total score (OR 1.06; *p* = 0.006), and disease duration (OR 1.16; *p* = 0.043) were the potential risk factors of female sexual problems by forward stepwise binary logistic regression.

**Conclusion:**

The present study reported that female sexual problems was more common in Chinese SLE female patients compared to controls. Having child, age, DMARDs usage, fatigue, and disease duration had great impacts on female sexual problems in Chinese SLE patients. Rheumatologists and nurses should pay close attention to SLE female patients’ sexual problems, especially those having no child, older age, not using DMARDs, fatigue, or long disease duration by health education or other methods to improve their sexual problems, and ultimately improve SLE patients’ quality of life.

## Introduction

Systemic lupus erythematosus (SLE) is an autoimmune inflammatory disease that predominantly affects women between puberty and menopause [1, 2]. SLE patients can suffer from clinical manifestations including arthritis, serositis, nephritis, rashes, scars, depigmentation, skin dimpling, hair loss, or neuropsychiatric problems [3], which have great impacts on quality of life (QoL) in this population [4]. Sexual problem is an important aspect of QoL and it is of great importance for SLE patients because it occurs predominantly in women with female to male ratio of 9:1 [5]. However, it is interesting to find that few SLE patients are willing to discuss their sexual problems with others, and most Chinese researchers or clinicians are reluctant to screen patients for sexual problems because they don’t think it’s their responsibility [6, 7]. What is more, only a limited number of studies related to SLE patients’ sexual problems have been conducted in China considering the conservative Chinese culture. Therefore, it is important to explore the rate and risk factors of sexual problems among Chinese SLE patients.

A recent meta-analysis [8] revealed the rate of sexual dysfunction (SD) in SLE patients, ranging from 15% to 85.9%, which indicated the severity of the poor assessment and management of these issues. Over the past several years, only a handful of studies [9–12] used a validated tool of the Female Sexual Function Index (FSFI) to explore the relationship between SLE and sexual problems, demonstrating that SLE was associated with an increased risk of female sexual problems. Tseng et al. [13] have reported that 52.5% have sexual problems in Taiwan SLE patients. Besides, Shen et al. [7] suggested that there were significant differences in sexual problems and relationship using a self-reported scale between Chinese SLE patients and healthy individuals. However, the rate and risk factors of female sexual problems using the FSFI in Chinese mainland SLE patients have remained unknown.

Previous studies [9–12, 14] have found that several factors were associated with female sexual problems in SLE patients, such as age, number of children, marital satisfaction, economic status, pain, disease duration, disease activity, certain psychological problems like depression and anxiety, functional status, and quality of life. Other factors such as higher dose of steroids also possibly result in female sexual problems [12]. Nevertheless, only one study has included fatigue factors in this topic [12]. Fatigue is one of the most common manifestation in SLE, which is described as a subjective feeling of tiredness and a lack of energy [15]. It is a multidimensional construct, wherein a distinction can be made between physical and mental fatigue. Previous studies reported that fatigue is significantly associated with a poor quality of life in SLE [16, 17], but the association between fatigue and female sexual problems is largely unclear. However, fatigue were closely associated with female sexual problems in other diseases [18, 19], it is therefore for us to hypothesize that fatigue has effects on female sexual problems in SLE patients.

Hence, the current study examines the independent association of fatigue with female sexual problems in a Chinese population. Moreover, we aimed to investigate the effects of SLE on Chinese mainland female patients’ sexual problems compared with healthy subjects, in order to provide a preliminary analysis of the clinical parameters, disease activity, and psychological parameters associated with sexual problems in SLE patients.

## Methods

### Participants

A total of 128 SLE patients were consecutively invited to participate in a single-center cross-sectional study. All data were collected consecutively by face-to-face questionnaires from January 2021 to December 2021 at the Ruijin Hospital, Shanghai Jiao Tong University School of Medicine. SLE Patients were included based on the following: (1) met the 2012 American College of Rheumatology diagnostic criteria [20], (2) were aged ≥ 18 years, (3) completed the questionnaire, (4) they had no comorbidities (e.g., serious infections or cardiac, respiratory, gastrointestinal, neurological, or endocrine diseases) that could influence SLE activity, (v) without any cognitive impairments and able to express their own thoughts in Mandarin freely.

Healthy subjects were selected from a population attending for an annual examination. A total of 121 convenience sample of women employees aged ≥ 18 years were recruited in 2020–2021. Control subjects were excluded if they exhibited current or history of other systemic diseases or psychiatric disorders. All eligible employees were distributed with an envelope containing a questionnaire composed of the FSFI, sociodemographic data, and a checklist of comorbidities by the assistant of each unit. It has high internal consistency of the sample in this study. All participants signed an informed consent, and this study was approved by the Survey Ethics Committee of the first author’s affiliated institution. All authors had full access to all data and have no competing interests.

### Demographic and clinical characteristics

Demographic and clinical data included age (years), BMI, marital status, having child, education, employment status, income/person/month (yuan), health insurance, religious beliefs, residence, history of hospitalization, history of family, comorbid condition, SLE disease duration (years), VAS pain (range 0–10), and use of DMARDs, corticosteroid, and biologics were obtained by viewing medical records combined with SLE patients’ self-report. At the same time, we used the Systemic Lupus Erythematosus Disease Activity Index (SLEDAI) to measure disease activity when collecting questionnaires [21].

### Assessment of sexual problems

Sexual problems were measured by a Chinese translation of the FSFI, a 19-item self-questionnaire that evaluates the female sexual problems in six domains: desire, arousal, lubrication, orgasm, satisfaction, and pain; and this tool is valid only in women who have active sexual life in the last month. The Cronbach’s alpha of the Chinese version was 0.91 and this is a reliable and valid instrument for the Chinese population [22]. Women who reported having no sexual activity in the past 3 months or had a score of zero in any domain of it were considered sexually inactive and were removed from the analyses concerning the FSFI scores. Summation of the six domain scores yields a total score (range 2–36) and a total score of < 26.55 is proposed as a criterion for impaired sexual function [23].

### Assessment of fatigue

Fatigue was assessed using the Chinese version of the multidimensional fatigue inventory (MFI) [24]. The MFI is a 20-item self-report instrument designed to measure fatigue. It covers the following dimensions: General Fatigue, Physical Fatigue, Mental Fatigue, Reduced Motivation and Reduced Activity. The total score of MFI-20 ranges from 20 to 80. The MFI-20 score indicates an individual’s fatigue degree; a high total score indicates serious fatigue. The internal consistency of the Chinese-version MFI-20 assessed by Cronbach’s alpha was high (> 0.8), which indicated that the MFI-20 was a reliable and valid instrument for assessing fatigue in Chinese patients.

### Assessment of depression

The patient health questionnaire-9 (PHQ-9) was used in the present study [25]. The PHQ-9 was based on the diagnostic criteria for depression from the Diagnostic and Statistical Manual of Mental Disorders, 4th Edition (DSM-IV). The scores for each PHQ-9 item range from 0 (not at all), to 1 (several days), 2 (more than half of the days), and 3 (nearly every day). A two-week recall period was used. The total score ranged from 0 to 27, with a higher score indicating greater self-reported depression. The internal consistency of the PHQ-9 in Chinese populations, assessed by Cronbach’s alpha, was 0.85 in this study.

### Assessment of quality of life

The 12-item short-form health survey questionnaire (SF-12) [26] is a generic QoL questionnaire that consists of 12 items that can be divided into 8 domains: physical functioning (PF), role limitations due to physical problems (RP), bodily pain (BP), general health perceptions (GHP), vitality, social functioning (SF), role limitations due to emotional problems (RE), and general mental health (GMH). The scores for the physical and mental composite summaries (PCS and MCS) were subsequently calculated based on the above domains. The scores in each domain could range from 0 to 100, and higher scores indicated better QoL. We used Cronbach’s alpha to assess the internal consistency of the SF-12 in this study, and the result was 0.88.

### Statistical analysis

Statistical analysis was performed using the IBM SPSS version 20.0 software. For continuously and normally distributed variables, we used mean ± standard deviation and independent samples t-test group to recognize differences between groups. For categorical variables, we used frequencies (%) and the group differences were measured by Chi-square test. Variables shown to be significant in the independent sample t-test, or chi-square test were included in the multivariate analysis using forward stepwise logistic regression model. The demographic, clinical and psychological characteristics, fatigue or quality-of-life parameters were defined as independent risk factors which were included in the model if *p* < 0.05 or removed if *p* > 0.10 according to forward selection technique. Statistical significance was considered when *p* < 0.05 (two-sided).

## Results

### Patient characteristics

**Fig. 1:**
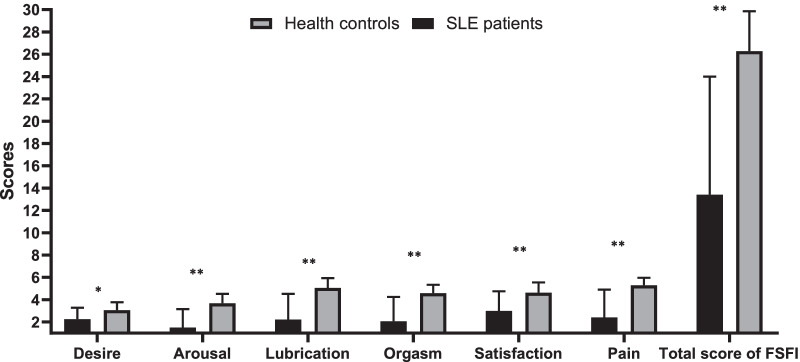
Comparison of the subscale and total scores of FSFI in SLE patients and controls. *P < 0.05; **P < 0.01

Table 1 shows the socio-demographic data of patients and controls. A total of 128 SLE patients and 121 healthy women were included. There were no significant differences between the baselines in two groups such as age, BMI, marital status, having child, educational, employment status, average monthly income, health insurance, religious beliefs and residence (*p* > 0.05). Figure 1 shows the subscale and total scores of FSFI between SLE and control groups. There were statistically significant differences between two groups including the subscale score of FSFI and the total score (*p* < 0.05).

**Table 1:** Demographic characteristics in SLE patients and health controls

Variables	SLE patients (n = 128)	Health controls (n = 121)	*P*
Age (years)	43.65 ± 7.13	43.59 ± 6.57	0.944
BMI (kg/m^2^)	23.12 ± 4.15	23.06 ± 3.35	0.902
Marital status			0.784
Single/divorced/widowed	31 (24.2)	28 (23.1)	
Married	97 (75.8)	93 (76.9)	
Having child (yes)	92 (71.8)	88 (72.7)	0.787
Education			0.992
Primary and below	25 (19.5)	23 (19.0)	
Secondary	30 (23.4)	29 (24.0)	
Graduate and above	73 (57.1)	69 (57.0)	
Employment status			0.379
Unemployed	64 (50.0)	71 (57.8)	
Employed	64 (50.0)	50 (42.2)	
Income/person/month			0.678
≤ 3000 Yuan	2 (1.6)	2 (1.7)	
1000–3000 yuan	28 (21.9)	24 (19.8)	
3000–5000 yuan	35 (27.3)	33 (27.3)	
≥ 5000 yuan	63 (49.2)	62 (51.2)	
Health insurance (yes)	109 (85.2)	102 (84.3)	0.756
Religious beliefs (yes)	17 (13.3)	11 (9.1)	0.262
Residence			0.145
Urban	114 (89.1)	100 (82.6)	
Rural	14 (10.9)	21 (17.4)	

Data are presented as mean ± SD or number (%)

*SLE* Systemic Lupus Erythematosus, *BMI* Body mass index

### Differences between non-sexual problems patients and sexual problems in SLE females

Table 2 represents the differences between SLE patients with or without sexual problems. Obviously, females with sexual problems had older age, longer disease duration, higher disease activity, higher depression level, higher fatigue level from both the total and subscale scores in MFI questionnaire, and lower level of quality of life, with a trend toward higher rate of unmarried, having no child, and DMARDs usage (*p* < 0.05).

**Table 2:** The comparison between SLE patients with and without sexual dysfunction concerning the demographic and clinic characteristics, and the different indices

Variables	With sexual dysfunction (n = 101)	Without sexual dysfunction (n = 27)	*t/F/x*^*2*^	*P*
Age (years)	40.90 ± 13.50	36.37 ± 6.84	2.44	0.018*
BMI (kg/m^2^)	23.14 ± 4.28	23.02 ± 3.69	0.13	0.896
Marital status			10.94	0.001**
Single/divorced/widowed	30 (29.7)	1 (3.7)		
Married	70 (70.3)	26 (96.3)		
Having child (yes)	68 (67.3)	24 (88.9)	4.90	0.027*
Education			0.01	0.973
Primary and below	20 (19.8)	4 (14.8)		
Secondary	29 (28.7)	9 (33.3)		
Graduate and above	52 (51.5)	14 (51.9)		
Employment status			1.17	0.279
Unemployed	53 (52.5)	11 (40.7)		
Employed	48 (47.5)	16 (59.3)		
Income/person/month			0.78	0.379
≤ 1000 Yuan	2 (2.0)	0 (0)		
1000–3000 yuan	23 (22.8)	5 (18.5)		
3000–5000 yuan	28 (27.7)	7 (25.9)		
≥ 5000 yuan	48 (47.5)	15 (55.6)		
Health insurance (yes)	86 (85.1)	23 (85.2)	0.01	0.996
Religious beliefs (yes)	13 (12.9)	4 (14.8)	0.07	0.792
History of hospitalization (yes)	81 (80.2)	20 (74.1)	0.48	0.488
History of family (yes)	14 (13.9)	1 (3.7)	2.13	0.145
Comorbid condition (yes)	62 (61.4)	12 (44.4)	2.51	0.113
Residence			0.01	0.974
Urban	90 (89.1)	24 (88.9)		
Rural	11 (10.9)	3 (11.1)		
SLE disease duration (years)	6.39 ± 7.22	3.80 ± 2.77	2.89	0.005**
VAS pain (range 0–10)	2.00 ± 1.50	1.67 ± 1.14	1.07	0.286
SLEDAI	6.28 ± 7.88	2.78 ± 3.64	3.33	0.001**
DMARDs usage (yes)	73 (72.3)	26 (96.3)	7.01	0.008**
Corticosteroid usage (yes)	84 (83.2)	23 (85.2)	0.01	0.802
Biologics usage (yes)	18 (17.8)	8 (29.6)	1.84	0.176
Depression (PHQ9)	5.27 ± 4.88	2.81 ± 4.54	2.35	0.020*
Fatigue (MFI)				
General fatigue	12.12 ± 3.09	10.67 ± 3.36	2.13	0.035*
Physical fatigue	12.37 ± 3.53	10.67 ± 2.98	2.29	0.024*
Mental fatigue	10.42 ± 3.67	8.12 ± 3.54	2.83	0.005**
Reduced motivation	9.35 ± 2.87	8.56 ± 3.30	1.23	0.220
Reduced activity	11.67 ± 3.03	9.96 ± 4.00	2.01	0.046*
Total score	56.43 ± 13.05	48.04 ± 13.51	2.95	0.004**
Quality of life (SF-12)				
PCS	44.11 ± 8.85	47.48 ± 7.91	− 1.80	0.075
MCS	45.96 ± 5.77	46.39 ± 4.82	− 0.36	0.720
Total score	90.06 ± 9.29	93.87 ± 8.07	− 1.94	0.046*

Data are presented as mean ± SD or number (%)

*SLE* Systemic Lupus Erythematosus, *BMI* Body Mass Index, *VAS* Visual Analog Scale, *SLEDAI* Systemic Lupus Erythematosus Disease Activity Index, *DMARDs* Disease-Modifying Anti-rheumatic Drugs, *PDD* Perceived Devaluation-Discrimination, *PHQ-9* Patient Health Questionnaire-9, *MFI* Multiple Fatigue Inventory, *SF-12* Short Form 12 health survey, *PCS* physical components summary, *MCS* mental components summary

*P < 0.05; **P < 0.01

### Determinants of sexual problems in SLE females

As shown is Table 3, stepwise logistic regression analyses were used to identify a model to predict SLE patients who would have female sexual problems. The results indicated that having child (OR 0.21; *p* = 0.024), age (OR 1.11; *p* = 0.002), DMARDs usage (OR 0.04; *p* = 0.004), MFI total score (OR 1.06; *p* = 0.006), and disease duration (OR 1.16; *p* = 0.043) were the potential risk factors of female sexual problems in SLE.

**Table 3:** Result of analysis of forward stepwise ordered logit regression models in SLE patients

Variables	B	SE	*P*	Exp (B)	95% CI	
					Lower	Higher
Step 1						
MFI total score	0.05	0.02	0.005**	1.05	1.01	1.09
Step 2						
DMARDs usage	− 2.36	1.05	0.025*	0.10	0.01	0.75
MFI total score	0.05	0.02	0.005**	1.05	1.01	1.09
Step 3						
DMARDs usage	− 2.58	1.06	0.015*	0.08	0.01	0.61
MFI total score	0.04	0.02	0.026*	1.04	1.01	1.08
Disease duration	0.12	0.06	0.035*	1.13	1.01	1.26
Step 4						
Having child	− 1.45	0.63	0.031*	0.19	0.06	0.97
DMARDs usage	− 2.88	1.11	0.009**	0.13	0.01	0.87
MFI total score	0.05	0.02	0.005**	1.07	1.00	1.12
Disease duration	0.13	0.06	0.039*	1.14	1.02	1.30
Step 5						
Age	0.11	0.04	0.002**	1.11	1.04	1.19
Having child	− 1.57	0.70	0.024*	0.21	0.05	0.81
DMARDs usage	− 3.28	1.16	0.004**	0.04	0.01	0.36
MFI total score	0.06	0.02	0.006**	1.06	1.02	1.10
Disease duration	0.15	0.07	0.043*	1.16	1.00	1.34

*SLE* Systemic Lupus Erythematosus, *DMARDs* Disease-Modifying Anti-rheumatic Drugs, *MFI* Multiple Fatigue Inventory, *OR* odds ratio, *CI* confidence interval

*P < 0.05; **P < 0.01

## Discussion

As is known to us, this is the first study investigating the prevalence and potential risk factors (e.g. fatigue, depression, disease activity) of female sexual problems using the FSFI in SLE patients from mainland China. The prevalence of female sexual problems in our SLE patients was 78.9%, higher than that reported in other studies using the FSFI (García et al. reported 45.9% [10], Cheng et al. 52.6% [13], and Serna-Peña et al. 28% [11]), which could be explained by the existence of conservative Asian culture and the different participants included in different studies with either Chinese or Western cohorts. All of these findings highlighted that female sexual problems should be included as part of the routine care for detection and management.

Previous studies [10–14] have reported that many reasons may lead to female sexual problems such as demographic characteristics, psychological problems, disease activity and drug usage etc. Our univariate analysis of demographic factors was consistent with previous studies showing that patients with female sexual problems had older age, no child, and poor marital status. For other clinical, psychological and quality of life variables, we found that patients with female sexual problems had longer disease duration, higher disease activity, DMARDs usage, higher levels of depression and lower sores of SF-36. It may be concluded that patients with poor physical and psychological functions may have difficulty engaging in daily life, including sex life.

In our study, except demographic, psychological and clinical factors that may lead to female sexual problems, we also included fatigue which was rarely reported [12]. Fatigue is a complex and multi-faceted phenomenon, defined as a feeling of physical tiredness and lack of energy [15]. Fatigue is important because it is a very common symptom of SLE, and significantly impairs patients' quality of life. Previous study has found that fatigue using the Multidimensional Fatigue Inventory (MFI-20) was associated with female sexual function in primary Sjögren's syndrome patients [17]. Therefore, it would be interesting to assess the effects of fatigue using the MFI-20 on female sexual problems in SLE. The results of our univariate analysis indicated that both total and almost subscale scores in MFI questionnaire were associated with female sexual problems, which was in accordance with the study by Pinto B et al. [12] stating that fatigue had a significant effect on female sexual problems. However, we found that reduced motivation was not associated with the female sexual problems in this study, which might be explained that SLE patients with reduced motivation tend to focus on their sex life and have good sexual function in daily life. Furthermore, this study revealed SLE patients with poor quality of life tended to suffer from female sexual problems. This study is the first to examine the quality of life and its impact on female sexual problems in Chinese SLE patients.

To identify which variables were most significantly correlated with female sexual problems, a stepwise logistic regression analysis was used. Only independent variables individually associated with FSD with a *p* value < 0.05 were entered into a stepwise logistic regression model. We found that having child, age, DMARDs usage, MFI total score, and disease duration were significantly associated with the FSD in SLE, which indicated that without child, older age, not using DMARDs, higher fatigue level, and longer disease duration were independent risk factors for female sexual problems in SLE. Measuring sexual function should be considered a vital part of the comprehensive evaluation of the health status of SLE patients, especially in those patients with older age, not using DMARDs, higher fatigue level, and longer disease duration.

There are, however, additional important shortcomings in this study that need to be addressed. First, the sample size was relatively small and the single-center study design might mean that results were not necessarily generalizable to a broader population. Another limitation of this study was the majority of patients were outpatients, therefore, our sample was not representative of the Chinese RA population. Second, previous study [7] has reported that body-image disturbance is associated with an impaired partner relationship in women with SLE, however, this emotional aspect was not measured in this study. Third, no causal conclusions could be inferred because the study was cross-sectional in design. Further prospective studies with expanded sample sizes should be conducted to support the development of effective interventions to improve sexual function of SLE patients. As is known to us, SLE is an autoimmune inflammatory disease, and autoimmune dysregulation plays an important role in inflammatory process. Previous study indicated that there is a potential link between SLE low androgen level and inflammation, and sex steroid play a role in modulating anti-inflammatory response not only in male but also in the female counterpart [27–29]. Therefore, another important issue is related to the lack of blood androgens assessment in this study. Indeed previous study clearly showed that and serum levels of testosterone was significantly lower in SLE patients than control [30], and low androgen was associated with fatigue [31, 32] and female sexual dysfunction in previous study [33]. A global consensus statement has recommended the treatment with testosterone in this kind of patients with sexual concerns [34]. Therefore, low androgen level could be the under investigated link between sexual concerns/fatigue and SLE, and testosterone treatment could be a therapeutic option in future study [35].

In conclusion, SLE considerably had sexual problems compared to controls in the present study. Older age, not using DMARDs, higher fatigue level, and longer disease duration had great impacts on female sexual problems in Chinese mainland SLE patients. Rheumatologists and nurses should pay close attention to SLE female patients’ sexual problems, especially those not having child, older age, not using DMARDs, fatigue, or long disease duration by health education or other methods to improve their sexual problems, and ultimately improve SLE patients’ quality of life.

## Data Availability

The datasets used and/or analyzed during the current study available from the corresponding author on reasonable request.
